# Marsupial Genome Sequences: Providing Insight into Evolution and Disease

**DOI:** 10.6064/2012/543176

**Published:** 2012-11-25

**Authors:** Janine E. Deakin

**Affiliations:** Division of Evolution, Ecology and Genetics, Research School of Biology, The Australian National University, Canberra, ACT 0200, Australia

## Abstract

Marsupials (metatherians), with their position in vertebrate phylogeny and their unique biological features, have been studied for many years by a dedicated group of researchers, but it has only been since the sequencing of the first marsupial genome that their value has been more widely recognised. We now have genome sequences for three distantly related marsupial species (the grey short-tailed opossum, the tammar wallaby, and Tasmanian devil), with the promise of many more genomes to be sequenced in the near future, making this a particularly exciting time in marsupial genomics. The emergence of a transmissible cancer, which is obliterating the Tasmanian devil population, has increased the importance of obtaining and analysing marsupial genome sequence for understanding such diseases as well as for conservation efforts. In addition, these genome sequences have facilitated studies aimed at answering questions regarding gene and genome evolution and provided insight into the evolution of epigenetic mechanisms. Here I highlight the major advances in our understanding of evolution and disease, facilitated by marsupial genome projects, and speculate on the future contributions to be made by such sequences.

## 1. Introduction

The class Mammalia is divided into three major lineages that last shared a common ancestor approximately 161 to 217 mya [1]. The egg laying monotremes (subclass Prototheria) represent the earliest offshoot of the mammalian lineage and possess a mixture of reptilian and mammalian features. Metatherians (marsupials) are the closest relatives of the more commonly known eutherian (“placental”) mammals, having diverged from them between 143 and 178 million years ago (mya) [1, 2]. The deep divergence of these three groups is especially valuable for providing insight into evolution amongst members of this class.

The unique features of marsupials have intrigued biologists since they were observed by European explorers, first in the Americas and later in Australasia. Perhaps their most recognisably distinct feature is their mode of reproduction. After a short gestation, marsupials give birth to altricial young that typically develop in a pouch or marsupium which is the feature responsible for the name of this infraclass of mammals. Marsupial evolution has placed a greater emphasis on development *ex utero* in the presence of a sophisticated lactation system characterised by changes in milk composition to meet the different nutritional requirements as the young develops [3]. The hindlimbs and eyes are poorly developed in marsupial neonates and most of the development of the brain, reproductive system, immune system, and endothermic regulation occurs postnatally [4]. In contrast, development of the more commonly studied eutherian mammals takes place largely *in utero*, in the presence of a well-developed and more invasive placenta than that found in most marsupials. 

There are over 300 extant species of marsupials distributed between the Americas (Ameridelphia) and Australasia (Australidelphia). The Ameridelphia consists of 99 species belonging to just two orders, whereas the Australidelphia are a diverse group of marsupials represented by 235 species belonging to five orders. The Ameridelphia and Australidelphia diverged from a common ancestor approximately 80 million years ago [5, 6], making comparisons between these two lineages similar in evolutionary terms to the comparison of human and mouse. 

For many years, research on marsupial genetics and genomics lagged behind that of their eutherian counterparts. However, rapid progress has been made since the sequencing of the first and subsequent marsupial genomes. After much debate in the late 1980s, three species were chosen as model marsupial species for comparative genetic and genomics studies, with one Ameridelphia species (*Monodelphis domestica—*South American grey short-tailed opossum), and two distantly related Australidelphia species, the tammar wallaby (*Macropus eugenii*) and the fat-tailed dunnart (*Sminthopsis crassicaudata*). These species were chosen for their ability to be easily bred in captivity and the availability of pedigreed colonies [7]. Several factors have contributed to the replacement of the dunnart species with another member of the Dasyuridae family, the Tasmanian devil (*Sarcophilus harrisii*), including the cessation of genetic research on the dunnart [8] and the emergence of the devastating devil facial tumour disease (DFTD) making genomics studies on devil of critical importance [9]. 

**Figure 1:**
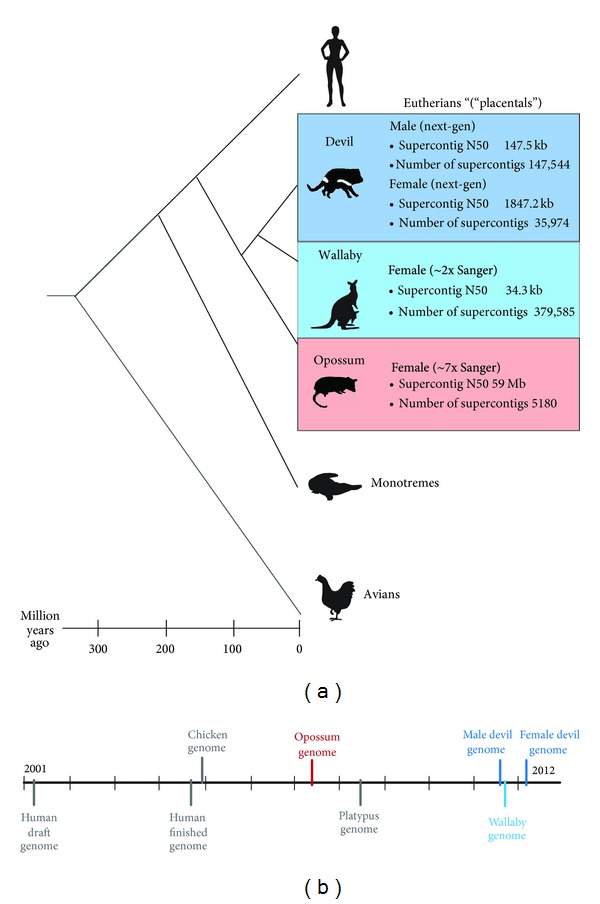
(a) The phylogenetic relationship of model marsupial species and a summary of their genome projects. (b) A timeline of the publication of marsupial genome projects and those of outgroups (human, platypus, and chicken) commonly used for comparative studies.

The genomes of all three model species have now been sequenced (Figure 1) [10–13] and have provided valuable insight into the evolution of mammalian genes and genomes, sex chromosomes, and epigenetic mechanisms. Furthermore, sequence of the devil genome and its facial tumour is helping us to gain an understanding of this unusual transmissible cancer [9]. These genome sequences are just the start, with many more marsupial genomes set to be sequenced in the near future [14]. These sequences could prove to be particularly valuable for species conservation as we begin to observe the effects of human activity on many already threatened marsupial species. 

## 2. Marsupial Genome Projects

Marsupial genomes are of a similar size as those of their eutherian counterparts but are typically packaged into several very large chromosomes. Cytogenetic studies have shown that marsupial karyotypes, ranging from 2*n* = 10/11 to 2*n* = 32, have changed very little throughout their evolution [15]. Sequencing of marsupial genomes has kept abreast with the latest advancements in sequencing technology, with a move from the more traditional whole genome shotgun approach using Sanger sequencing [13] to being sequenced entirely by next generation technology [11, 12]. The different approaches used have resulted in differences in genome assembly quality. Additional resources, such as transcriptome sequence as well as physical and linkage maps, have proven valuable for overcoming some of the limitations in sequence coverage depth. Marsupial genome sequences have not provided all the answers but have established the foundation for more focused efforts on particular questions. 

### 2.1. The Opossum Genome

The opossum was the chosen species for the first marsupial genome project. The opossum is a laboratory marsupial, being able to be bred in captivity in a similar manner to laboratory mice [16]. This species has been used as a model for biomedical research, particularly as an animal model for UV-induced melanoma [17], and has the added advantage of being raised for genetics research in pedigreed colonies for over 30 years [18]. These colonies have been used for the construction of linkage maps [19, 20], which represent valuable resources for correlating phenotypic variation with genomic sequence. 

The genome of a partially inbred female opossum was sequenced to a depth of almost sevenfold coverage using a whole genome shotgun approach with traditional Sanger sequencing [13]. The high quality of the genome assembly is reflected in the assembly statistics, with 5180 scaffolds and a scaffold N50 of 59.8 Mb (a measure of assembly quality representing the length of the scaffold at which 50% of scaffolds in the assembly are shorter). This makes it the best assembled marsupial genome (Figure 1). Most of this sequence (97%), contained in 216 scaffolds, has been anchored onto the eight opossum autosomes and the X chromosome by cytogenetic mapping of BAC (Bacterial Artificial Chromosome) clones corresponding to the ends of scaffolds [21]. Approximately 19,000 protein-coding genes have been annotated by the Ensembl consortium in the MonDom 5.0 assembly (http://www.ensembl.org/). In the absence of transcriptome data, these annotations were based largely on comparisons with genes from distantly related species such as human [22] and hence afford reliable gene annotations only for well-conserved genes. More divergent genes have either failed to have been identified or have been misannotated [23]. However, the recent sequence of transcriptomes of 26 different opossum tissues (http://www.opossumbase.org/?q=transcriptome) will lead to more accurate annotations of opossum genes and more importantly, the identification of novel transcripts. 

### 2.2. Sequencing of the “Kangaroo” Genome

In 2004, the National Human Genome Research Institute recognized the value of sequencing a second marsupial genome and provided partial funding for the sequencing of the tammar wallaby genome, with matched funding provided from Australian sources. The tammar wallaby, a member of the kangaroo family Macropodidae, boasts a number of unusual attributes, including the longest period of embryonic diapause [24], highly synchronized breeding, and a complex lactation system where mothers can produce two different types of milk at the same time, with one teat producing milk suitable for a neonate and an adjacent teat delivering milk appropriate for the developmental needs of a young-at-foot [25]. For these and many more reasons, the tammar wallaby has been the most intensively used marsupial for studies on genetics, reproduction, development, and physiology.

Unfortunately, funding for this Australian marsupial did not extend to sequencing it to the same depth as the opossum, resulting in only twofold sequence coverage by Sanger sequencing of a female tammar wallaby genome. Improvements to the original Meug_1.0 genome assembly were made by the incorporation of ABI SOLiD paired-end sequence data (Meug_1.1), as well as Roche 454 data for 0.3x coverage and 5x coverage by paired Illumina read (Meug_2.0) [10]. The resulting Meug_2.0 assembly consists of over 300,000 scaffolds with an N50 of just 34.3 kb. Assigning so many scaffolds to chromosomes was not feasible using the same approach as that used for anchoring the opossum genome. Instead, a virtual map of the genome was constructed [26] from integrating the available tammar wallaby cytogenetic [27, 28] and linkage maps [29]. This approach allowed just 6% of the genome to be assigned to the seven wallaby autosomes [10]. The genebuild, performed by Ensembl on the Meug_1.0 assembly, resulted in the annotation of 15,290 protein-coding genes projected from high-quality reference genomes of distantly related species [10]. Gaps are common within these annotated genes. Transcriptomes from six different tissues have been sequenced [10, 30, 31] permitting some of the limitations of this lightly sequenced genome to be overcome. 

### 2.3. Tasmanian Devil Genome Sequencing Projects

There have been two independent genome sequencing projects for the Tasmanian devil, spurred on by the devastating transmissible cancer threatening the Tasmanian devil population with extinction [32]. The main aim of these projects was not necessarily to obtain a well-assembled and annotated genome assembly, but to identify sequence variants that may potentially confer resistance or at least delay the onset of the disease [12] and provide the first step towards identifying the mutations present in the DFTD genome [11, 12]. 

The first genome project sequenced DNA from two male devils from different locations and with suspected differences in DFTD susceptibility with the assumption that genetic variants detected between these two individuals may be responsible for the difference in response to DFTD [12]. A combination of different next generation sequencing platforms was used. Sequencing data from both males was combined to produce a genome assembly consisting of *∼*140,000 scaffolds and an N50 of 147.5 kb, which is better than the tammar wallaby genome assembly. Genes were not actually annotated in this assembly but exons identified in the opossum genome were used to identify exons in the devil in order to detect sequence variants causing amino acid differences between the two individuals [12]. 

The second genome project sequenced a female devil using the Illumina platform to sequence both short and large insert libraries, which were assembled into ~35,000 scaffolds with an N50 of 1.8 Mb [11], a vast improvement over that obtained by the previous genome project. Sequencing of chromosomes sorted by flow cytometry has enabled the assembly to assign 99% of scaffolds to chromosomes; the accuracy of which has been attested by cytogenetic mapping [11]. Gene order has been inferred based on the opossum assembly [11], but caution is warranted using this gene order for comparative analysis as considerable rearrangement between the opossum and devil has been observed from gene mapping [33]. It should also be noted that the DNA used for sequencing was obtained from a fibroblast cell line, which displayed a trisomy for chromosome 6 [11] and raises concern that other mutations may have arisen in culture. Transcriptome sequence from 12 pooled devil tissues assisted the annotation of genes using the Ensembl GeneBuild Pipeline, resulting in the identification of 18,775 protein coding genes of which ~1200 had no orthologue in the human or opossum genomes [11]. 

### 2.4. Major Research Areas Utilizing Marsupial Genome Sequences

Thus, there are four genome assemblies for three marsupial species. Each assembly has its own set of limitations but there is no doubt that they all provide a valuable resource for marsupial genomics research and a foundation from which more focused studies can be built. Some of the most prominent areas of research using marsupial genome sequences include the evolution of genes involved with (a) immunity, particularly in regards to those genes that may be contributing to the immunological protection of the altricial young, (b) the complex lactation system of marsupials, and (c) development of the young. Research into the evolution of marsupial sex chromosomes is an area that has been pursued for many decades, with marsupial genome sequences providing important insight into their evolution and the foundations necessary for unraveling the evolutionary origin of the remarkable epigenetic mechanism of X chromosome inactivation. Genomic imprinting is another epigenetic phenomenon that has received considerable attention in marsupials and again is an area that has greatly benefited from marsupial genome projects. Marsupial genome evolution has been of significant interest, not only in terms of the evolution that has occurred amongst marsupials, but also in determining the evolutionary events that have shaped mammalian genomes. Currently, one of the most urgent fields of research in marsupial genomics is devil facial tumour disease, as gaining a rapid understanding of this disease is of critical importance to the survival of the Tasmanian devil. 

## 3. Immune Genes

The identification of immune genes could prove critical for marsupial conservation programs. Characterisation of genes involved in the immune response will lead to a better understanding of the response of marsupials to disease, particularly with diseases such as DFTD in devils [32], a viral papillomatosis and carcinomatosis syndrome in western barred bandicoots (*Perameles bougainville*) [34], and *Chlamydia* in koalas (*Phascolarctos cinereus*) [35], threatening the survival of these species (or at least populations of these species). In addition, some of the unique features of marsupials have made discerning the evolution of immune genes of particular interest. For instance, the development of the immune system of marsupials occurs almost entirely after birth, when exposed to potentially pathogenic microorganisms [36–39], making characterisation of marsupial immune genes particularly interesting for determining how they survive. The highly specialised lactation system of marsupials also makes understanding the evolution of these genes biologically important [10]. Immune genes are also known to be the most rapidly evolving genes within the genome and are therefore also of interest from an evolutionary perspective.

### 3.1. Characterisation and Evolution of the Major Histocompatibility Complex

The Major Histocompatibility Complex (MHC) represents one of the most studied regions of the vertebrate genome, mainly due to its pivotal role in the immune response. It is a dynamically evolving region of the genome due to selective pressures posed from the host-pathogen arms race. 

The human MHC was sequenced over a decade ago as part of the human genome project and was found to be a large, gene-dense region spanning 3.6 Mb with 224 protein coding genes [40]. Sequencing of the mouse MHC revealed a similar overall organisation of the complex, which was divided into three regions to reflect the Class of MHC genes each contained. Genes encoding for the *α* chain component of Class I molecules are found in the Class I region, along with nonimmune genes termed framework genes that are conserved between human and mouse [41]. The Class II region consists of genes encoding for the alpha- and beta-chains of the Class II molecule as well as antigen processing genes such as *TAP *and *PSMB. *The Class III region does not contain genes encoding for MHC molecules but was so named simply because it separates the Class I and II regions. It is renowned for being the most gene-dense region of the genome, where genes involved in the complement, heat-shock, and inflammatory responses are located. Sequencing of the chicken MHC revealed an astonishingly different organisation. It was found to be considerably smaller, spanning only 92 kb, and containing a mere 19 genes [42]. The organisation is different too, with Class II and Class I regions adjacent [42]. Determining the MHC organisation for marsupials, which bridge the 200 million year phylogenetic gap between avians and eutherians, was critical for elucidating the evolution of this gene complex. 

**Figure 2:**
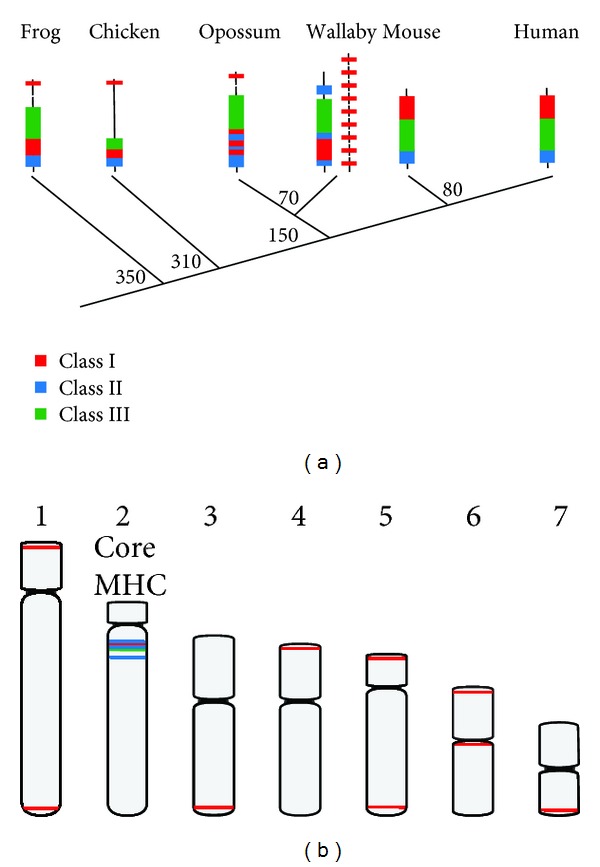
MHC organisation. (a) A comparison of MHC organisation across different vertebrate lineages. The organisation of the opossum MHC resembles the ancestral organisation observed in frog and chicken, and the organisation in human and mouse is derived (modified from [43]). (b) The tammar wallaby core MHC region is substantially different to that of the opossum, with Class II genes on either side of the Class III region and Class I genes distributed on different chromosomes.

Sequencing of the opossum MHC presented an opportunity to characterize the gene content and organisation of a marsupial MHC and was the first multimegabase region of the genome to be annotated. In size and complexity, the opossum MHC is similar to that of eutherian species, spanning 3.95 Mb and containing 114 recognisable genes [43]. However, gene arrangement is strikingly different, with a combined Class I/II region, also containing the antigen processing genes, and a well-conserved Class III region. This organisation led to the hypothesis that marsupials possess an ancestral MHC organisation, whereas Class I genes have relocated in eutherian species to form a distinct Class I region [43]. This hypothesis has since been supported by analysis of the *Xenopus tropicalis* MHC (Figure 2(a)) [44].

Although determining the organisation of the opossum MHC proved informative for elucidating the evolution of this region in vertebrates, comparisons of organisation and gene content have shown that the MHC is dynamic, evolving in response to pathogenic and environmental pressures [45, 46]. Hence, differences between marsupial species would not be unexpected. Characterising the MHC of the other sequenced marsupials was not possible with the patchy sequence coverage. Therefore, a focussed sequencing strategy was required. A BAC-based approach was used to sequence the MHC regions of the tammar wallaby and devil, revealing some unexpected surprises [47–49]. 

To sequence the tammar wallaby MHC, BAC clones containing genes from the different regions of the opossum MHC were isolated and initially cytogenetically mapped to chromosomes. From cross-species chromosome painting studies, it was established that MHC-containing chromosome 2 in the opossum is homologous to chromosome 2 in the tammar wallaby [50], and therefore, the chromosome on which the MHC would reside. Indeed, Class II, antigen processing genes, Class III, and genes flanking the opossum MHC were all located on wallaby chromosome 2. Surprisingly, none of the Class I containing BACs mapped to this same location but were found dispersed across the genome, mapping typically to the ends of every other autosome [51]. The subsequent isolation of many more BACs containing MHC genes and their sequencing revealed a core MHC on chromosome 2, which has a novel arrangement compared to that of other mammals (Figure 2(b)). Class I genes present in this region are limited to those with nonclassical functions (i.e., a function other than the presentation of peptide antigens) and the Class II regions have formed two distinct clusters that are separated by Class III genes (Figures 2(a) and 2(b)) [47]. Nine Class I genes, including as many as three with a predicted classical role, localised to other regions of the genome [48]. Intriguingly, fragments of Kangaroo Endogenous Retrovirus (KERV) sequence were discovered within the core MHC as well as adjacent to many of the dispersed Class I genes, suggesting a potential role of KERV in the rearrangement and movement of MHC genes [47, 48, 51]. 

The main driving force behind the characterisation of the devil MHC was DFTD and determining how this transmissible tumour is able to evade the immune response and if there are MHC allelic differences that may confer resistance to DFTD [49, 52]. Initially, extremely low levels of MHC diversity were believed to be responsible for enabling devil facial tumours (DFTs) to escape detection of the host's immune system since the MHC genotype of the tumour was so similar to that of host [52]. However, skin graft experiments demonstrated that devils do have the ability to distinguish between self and nonself, even in the presence of little or no variation in the MHC alleles [53]. Therefore, there must be some mechanism employed by DFTs to evade the immune response. Recently, it has been found that despite some Class I and II transcripts being expressed, MHC Class I protein is barely detectable on the surface of DFT cells [54]. Epigenetic manipulation and treatment with cytokines restore Class I protein levels in DFT cells, suggesting that epigenetic changes play a role in DFT immune evasion [54]. 

The devil MHC is yet to be as extensively characterised as those of the opossum or tammar wallaby but BAC-based sequencing has resulted in the annotation of a 960 kb region containing Class I and II genes [49]. Comparisons between the three sequenced marsupial MHC regions indicate that the organisation of the devil MHC may be more similar to that of the opossum than the tammar wallaby, suggesting that the very unusual dispersal of Class I genes observed in the wallaby is not a common feature of the Australian lineage.

One of the interesting features discovered within the devil MHC was the presence of three copy number variations between two individuals with varying immune responses to the disease; one that failed to mount an immune response (Spirit) and one that produced an antibody response to DFTD although eventually succumbed to the disease (Cedric). Perhaps the most noteworthy difference is a ~1.6 kb deletion in Cedric's MHC, resulting in a classical Class I gene becoming a pseudogene. At a population level, this deletion was most common in northwestern Tasmania, which is currently disease-free and at the disease front, and less prevalent in DFTD-affected areas, leading to the hypothesis that this deletion may have a significant impact on the immune response to DFTD [49]. More recently, a test of the frequency of this deletion in healthy and DFTD-affected individuals showed no significant difference in its occurrence between the two groups [55]. Thus, the difference in immune response to DFTD between Cedric and Spirit is unlikely to be due to this deletion. The focus of future studies into differences in DFTD susceptibility may need to move away from focussing solely on the MHC to examine variation at other immune gene loci [55]. 

### 3.2. The Marsupial Immunome

Claims were made from early studies that the immune response of marsupials was inferior to that of their eutherian counterparts [56, 57]. The amazing survival of marsupial young born without an adaptive immune system is truly remarkable and would indicate that marsupials are more than adequately able to immunologically protect themselves. The release of marsupial genome sequence has allowed the complexity of the marsupial immunome to be examined and determine whether this inferiority label is warranted. 

The human genome has over 800 genes vital to the immune response [58, 59]. A search for these in the opossum genome has revealed a similar level of complexity of the marsupial immunome as is found in human [60]. Among these genes are the highly divergent immune genes such as cytokines, natural killer cell receptors, and antimicrobials. As a consequence of low levels of sequence conservation across taxa, the isolation of many of these genes was difficult to impossible by standard laboratory-based methods [61] but the development of sophisticated bioinformatic approaches [23] has overcome this problem. Consequently, a database containing sequences for 538 expressed tammar wallaby immune genes and 1653 opossum immune genes (1639 predicted genes, 24 expressed) has been established [30].

Notably, antimicrobial genes have undergone unprecedented expansion and diversification, which may be linked to the protection of the highly altricial young. In particular, the cathelicidin gene family has 12 members in the opossum (human and mouse have only one) that show an extreme level of sequence heterogeneity, with as little as 1% sequence identity for mature peptides [60]. Annotation of cathelicidins in the tammar wallaby genome shows that they are just as diverse as those of the opossum, consisting of 14 genes and sharing ~28% sequence identity at the peptide level [62]. These cathelicidins, expressed in milk and young, are more highly expressed in the later stages of lactation and pouch young development than they are at birth [63, 64]. Although they act as highly potent antimicrobials [62, 63], other factors may be more important for conferring protection at the very early stages of pouch life. 

Poor mixed lymphocyte responses in marsupials were responsible for their T-cell-mediated response being labelled inferior [57]. This makes the discovery of a novel T-cell receptor gene (*TCRM*) in marsupials, in addition to the four traditional T-cell receptor genes (*TCRA, TCRB, TCRD, and TCRG*) found in eutherians and other species, all the more intriguing [65]. The function of *TCRM* is unknown at this stage but an orthologue of this gene has been recently identified in the platypus, suggesting that it evolved early in mammalian evolution and was subsequently lost in the eutherian lineage [66, 67]. 

Now that the identification of marsupial immune genes is well at hand, research should shift towards determining the functions of these genes, particularly for novel genes such as *TCRM. *As more transcriptome data becomes available, marsupial-specific immune genes that have failed to be identified using the current approach of searching with known human immune gene sequence may also be discovered. 

### 3.3. Immune Genes and Marsupial Conservation

Although home to the majority of the world's marsupials, Australia has a terrible reputation for extinction of its native species, recording the world's highest extinction rate for recent mammals [68]. In an attempt to counteract this situation, research has focused on the threats caused by increasing habitat fragmentation and predation from introduced species on Australia's unique marsupial populations [69]. However, the risks posed by emerging diseases are becoming increasingly apparent, with the threat of emerging diseases to wildlife proposed to increase as a result of climate change and human encroachment on wildlife habitat [70], which could have devastating effects on many already threatened marsupial species (Table 1). Understanding how the immune response of marsupials compares to that of their eutherian counterparts would help to determine if vaccines or treatments used to control the same or similar disease in other mammalian species are likely to be useful for marsupials.

Early studies on the immune response of marsupials reported distinct differences to those of eutherian species. In particular, the prolonged nature of their primary response lasting at least nine [71] to 26 weeks [72] contrasts dramatically with the short-lived primary response of eutherians. In the past, studies on the marsupial immune response were hampered by a lack of marsupial-specific reagents. Fortunately, recent advances in marsupial genomics and immunogenetics are able to rapidly fill knowledge gaps and are providing the information necessary for the development of marsupial-specific reagents required to accurately assess the immune response [61]. 

A loss of genetic diversity is a major concern for threatened marsupial species since many marsupials are restricted to offshore islands or exist in small isolated populations due to habitat fragmentation [73]. This increases the susceptibility of the health of a population if a novel disease is encountered [34, 74, 75]. Given that pathogens are known to drive genetic diversity at immune gene loci in their hosts, it would seem essential to assess the genetic diversity of immune genes. Selection is more likely to act on these loci to retain higher levels of genetic diversity than at random loci in the genome [76]. Typically, the only immune genes included in marsupial conservation studies have been Class I and/or Class II loci from the MHC. However, the assessment of diversity at other key immune loci may also be important for species conservation [77, 78]. Until now, this has never really been considered as an option for marsupials due to the difficulty in obtaining sequences for rapidly evolving immune genes with little sequence conservation to those in eutherians. Even measuring the diversity of MHC loci has been limited to just six species; the tammar wallaby [79], black-footed rock wallaby [80], the Tasmanian devil [52], the brushtail possum [81], the koala [82], and the western barred bandicoot [83]. Among this list, both the devil and bandicoot are endangered, have critically low MHC genetic diversity and disease affecting conservation efforts [52, 83]. 

Studies focusing exclusively on MHC allele diversity, such as those on the northern elephant seal or a feral herd of cattle, have found low levels of diversity in thriving populations with no obvious increase in disease susceptibility [84–86]. The opposite situation has also been found where a desert population of bighorn sheep with high levels of MHC diversity was suffering declining numbers due to infectious diseases [87]. In both situations, genetic diversity at other immune loci may be the critical factor determining disease susceptibility [78]. Supporting this idea, an examination of genetic variation in cytokines and other immune genes has linked variation within these genes in field voles to differences in immune responses to multiple parasites and identified signatures of putative pathogen-mediated selection which may be driving genetic diversity at a subset of immune gene loci [77, 88]. The affordable nature of next generation sequencing to obtain species-specific sequences now makes it possible to assess genetic diversity of more immune genes and to determine whether there is a link between immune gene variants, immune response, and overall animal health. Although next generation sequencing is yet to be used for this purpose in marsupials, the identification of marsupial immune genes and the establishment of a database [89] have been an important first step towards this objective. 

## 4. Lactation Genes

The reproductive strategy adopted by marsupials has resulted in a greater maternal investment in lactation than in *in utero* development. During lactation in marsupials, the composition of the milk produced by the mother progressively changes in composition of all major and minor components [90]. Marsupial lactation is divided into three phases based on the composition of the milk produced and the suckling status of the young [25]. In the tammar wallaby, the first phase is short, covering the period from late pregnancy to parturition. Phase two extends to day 200 of pouch life, where the milk produced is high in complex carbohydrates. During the first part of the phase (Phase 2A) the young is permanently attached to the teat (days 0–100), whereas the young suckles intermittently during the second half of this phase (Phase 2B). Phase 3 is associated with an increase in milk production and a change from a milk high in carbohydrate to one rich in protein and lipid. At this time, the young will be in and out of the pouch (days 200–300) [25]. Therefore, an examination of gene expression during lactation is likely to reveal signatures of evolution specific to the marsupial reproductive strategy. 

Mammary gland transcriptome analysis estimates that 10% of genes expressed in this tissue are marsupial specific [31]. Genes expressed in mammary gland can be divided into two groups; one group that is expressed from birth and throughout lactation and the other consists of genes expressed at specific stages of lactation [31]. Group 1 genes are common between eutherians and marsupials and includes genes encoding for *α*-, *β*-, and *κ*-caseins and *β*-lactoglobulin. Group 2 genes are more interesting as it includes 75 novel genes [31], such as those encoding for late lactation proteins (*LLPA *and *LLPB*) [91]. The functions of some of these genes were determined using *in vitro* assays, revealing their involvement in the inflammatory response, immune modulation, growth, and differentiation [92]. 

Among the genes of interest are those involved in the immunological protection of the young. *WFDC2*, a gene thought to play a role in innate immunity, is expressed during pregnancy, early lactation, and then is downregulated until the end of lactation and involution [93]. This correlates to stages of lactation associated with higher risks of infection [94–96]. The protein encoded by the *WFDC2* gene displayed antimicrobial activity against bacteria including *Staphylococcus aureus,* but not against the common enteric commensal bacteria *Enterococcus faecalis, *suggesting that this protein may provide immunological protection to the young against potentially pathogenic bacteria while maintaining the gut microbiota [93]. Similarly, the use of a marsupial-specific microarray to investigate differential gene expression has identified 47 genes upregulated during mammary gland involution. Among these are genes that could potentially play a protective role against mammary gland infection, such as *ABP1, C1QB, C4A,* and *CSF2R*β** [97].

Comparative analysis of genes encoding milk proteins has provided insight into their evolutionary origin. For instance, the evolutionary history of the three genes (*CSN1, CSN2,* and *CSN3*) encoding for the three different caseins has been unravelled [98]. These genes occur as a syntenic block of genes, with *CSN1* and *CNS2* located adjacent to one another and *CSN3* a greater distance away in all mammals. Additional casein genes are located between *CSN2* and *CSN3* in the platypus (*CSN2b)* and in eutherians (e.g., *CSN1S2*) but these appear to be absent in marsupials. This region in the opossum is enriched with transposon-like repetitive elements, suggesting that the ancestral gene from which *CSN2b* and *CSN1S2* evolved may have been lost in the marsupial lineage due to several rounds of transposition occurring in this region [98]. Another example is the early-lactation protein gene (*ELP*), which was initially proposed to be a marsupial-specific gene [31]. However, it has recently been shown that *ELP* and the eutherian colostrum trypsin inhibitor (*CTI*) evolved from a single gene present in the therian ancestor, most likely more closely resembling the *CTI* gene than marsupial *ELP *[99]. Continued comparative analysis of milk genes between all three major mammalian lineages will help to elucidate the origin of the sophisticated marsupial lactation system. 

## 5. Development Genes

In contrast to the rapidly evolving immune genes discussed previously, other gene families are highly conserved. One such family that has fascinated biologist is the *HOX *gene family responsible for the patterning of embryonic development. *HOX* genes occur as clusters on chromosomes and are remarkably organised in the order of expression and their role in development along the anterior-posterior body axis [100]. *HOX *genes are thought to have arisen from a single *HOX *gene that underwent tandem duplication. The four different chromosomal clusters present in vertebrates are most likely the result of two rounds of whole-genome duplication [101]. Comparisons of the coding and noncoding sequences in such a gene family are likely to reveal sequences that are essential to the proper functioning of these developmentally important genes, while at the same time revealing sequences associated with potentially lineage-specific gene regulation. 

*HOX* gene clusters were well annotated in the opossum genome but were dispersed over many scaffolds in the tammar wallaby sequence, making it necessary to sequence BACs spanning each of the four clusters. A high level of conservation between vertebrate species in sequence similarity of orthologous genes and gene arrangement within each cluster was observed. In addition, three long non-coding RNAs orthologous to those found in eutherians (*HOXA11AS, HOTAIRM1, and HOTAIR*) were identified in marsupials as well as five conserved microRNAs (*mir-10a, mir-10b, mir-196a, mir-196a2, and mir-196b), *suggesting an important regulatory role is performed by these genes [102]. A novel microRNA expressed in testis and fibroblast was also discovered [102]. 

Analysis of the role of *HOX *genes in marsupials is only just beginning. At birth, the forelimbs are well developed, having assisted the young on its climb into the pouch. The altricial hindlimbs present at birth undergo rapid growth and development in the pouch. In the case of the tammar wallaby, the hindlimbs show specialised development for a hopping mode of locomotion. This disparity between the development of the fore- and hindlimbs in marsupials presents an interesting case in which to study* HOX* gene expression. *HOXA13* and *HOXD13* are essential for digit formation in mice [103] and have been investigated in the tammar wallaby. Compared to chicken and mouse, *HOXA13* was transiently expressed in the tammar wallaby and detected at an earlier stage of fetal development in the forelimb than hindlimb [104]. *HOXD13* expression was much more conserved between these three species but once again, hindlimb expression commenced at a later stage than the forelimb [104]. Future comparisons of *HOX* gene expression will elucidate how morphological diversity is achieved from such highly conserved gene clusters. 

## 6. Sex Chromosome Evolution

On a broader scale, marsupial genome sequences have enabled the evolutionary history of particular chromosomes, such as the sex chromosomes, to be traced. Sex chromosomes have evolved multiple times during vertebrate evolution [105] as a result of a sex determining allele being acquired by one homologue of an ordinary pair of autosomes. Male- (or female for ZW system) advantage genes accumulate in a nonrecombining region, leading to progressive degradation of the chromosome [106]. Marsupial and eutherian mammals typically have XX females: XY males, where the X chromosome is a large, gene-rich chromosome and the Y has degraded into a small, gene-poor chromosome. Determining the gene content and arrangement on marsupial X and Y chromosomes has provided insight into the evolution of mammalian sex chromosomes and the epigenetic phenomenon of X chromosome inactivation, a mechanism believed to have evolved to equalise the expression of X-borne genes between males and females by silencing one X chromosome in females. 

### 6.1. Gene Content and Organisation of Marsupial X Chromosomes

After the discovery of X chromosome inactivation in eutherian mammals, Susumu Ohno predicted that the gene content of the X chromosome would be conserved among mammals as rearrangements with autosomes would risk disrupting the inactivation mechanism [107]. This prediction was confirmed by finding that the tammar wallaby X chromosome hybridised to two-thirds of the human X chromosome [108]. The remaining third of the human X appeared to be autosomal in marsupials [109]. Marsupial genome sequence enabled the fusion point of this addition to the eutherian X chromosome to be uncovered and provided information on gene content and arrangement of marsupial X chromosomes. 

The opossum X chromosome consists of ~442 protein-coding genes (Ensembl 68) and 302 of these have orthologues in the fragmented tammar wallaby assembly. This is far fewer than the ~1500 genes identified on the human X. A comparison of the human and opossum genomes exposed the border of the region conserved between eutherians and marsupials (X conserved region - XCR) and the region added in the eutherian lineage (X added region - XAR) to correspond to human Xp11.23, between *RGN* (located on opossum chromosome 7) and *RBM10* (located on opossum X chromosome) [13]. This fusion point was also confirmed in the tammar wallaby by gene mapping [28]. In the African elephant, a member of the most basal clade of eutherian mammals, the centromere appears to be positioned at this fusion point, suggesting that the eutherian X chromosome was the result of a Robertsonian fusion between the XAR and XCR [110]. 

In contrast to the well-conserved order of genes on the X chromosome in eutherian species [110–113], gene order between marsupials is highly rearranged [13, 28, 33]. Comparative analysis of the opossum and human X chromosomes identified at least 26 breakpoints [13]. The conservation of X-linked gene order in eutherians is supposedly a consequence of selection against rearrangements that could disrupt the spread of *XIST*, a long non-coding RNA critical to eutherian X inactivation, across the chromosome from the centrally located inactivation centre and affect the tightly controlled silencing mechanism [13]. In keeping with this idea, extensive searches failed to detect an opossum *XIST* [114]. Furthermore, genes flanking *XIST* in eutherians are adjacent in other vertebrates, but there is a breakpoint between these genes in marsupials [28, 115–117]. This places the evolution of *XIST* after marsupial/eutherian divergence. Conversely, genes corresponding to the eutherian XAR region show a high level of conservation in gene order between eutherians and marsupials [13, 28]. 

**Figure 3:**
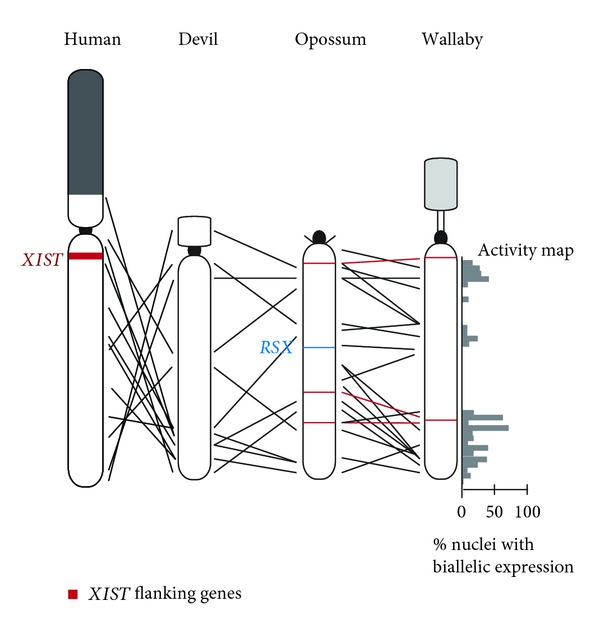
Comparative map of the human and marsupial X chromosomes. The position of *XIST, *genes flanking *XIST* in eutherians, and the recently discovered opossum *RSX* gene are indicated. The dark grey region on the human X corresponds to the region added to the X in the eutherian lineage (XAR). A heterochromatic region of the tammar wallaby X chromosome is indicated in light grey. An activity map is shown to the right of the tammar wallaby X chromosome. Grey bars indicate the percent of nuclei displaying biallelic expression, that is, expressed from both the active and “inactive” X chromosomes.

The extensive rearrangement of marsupial X chromosomes led to the suggestion that a marsupial-specific *XIST*-like gene was unlikely to be present in marsupials [28]. Astonishingly, such a gene has been found. Grant et al. [118] discovered *RSX* (RNA on the silent X) accidently when using a BAC (Bacterial Artificial Chromosome) clone encompassing the *HPRT1 *gene in RNA-FISH experiments designed to detect primary transcripts of a gene within interphase nuclei. They detected cloud-like signals more reminiscent of *Xist* signals detected in mouse ES cells than the discreet dot-like signals they had expected. The sequence causing this cloud-like signal corresponded to a 47 kb segment downstream of *HPRT1, *which forms a 27 kb mature non-coding RNA. Although *RSX* has no sequence homology to *XIST,* these two non-coding RNAs share a high GC content, an enrichment of tandem repeats within the 5′ region and conserved motifs potentially involved in the generation of stem-loop structures. Like *XIST, *expression is observed only in females and it coats the inactive X in *cis. *In addition, transgene experiments in mouse cells demonstrate that *RSX *is capable of inducing gene silencing. Orthologues of *RSX *orthologues were detected in Expressed Sequence Tag (EST) data for two Australian marsupials, the tammar wallaby and brushtail possum, supporting the proposal of *RSX* representing a marsupial-specific X inactivation centre [118]. The central position of *RSX* on at least the opossum X chromosome, again reminiscent of *XIST,* is also intriguing (Figure 3). Perhaps a central location is important for the spread of *RSX *along the chromosome. 

### 6.2. The Marsupial Y Chromosome

The Y chromosome has an exceptionally important function, playing a key role in sex determination and differentiation. In contrast to the conserved gene content for the XCR between marsupials and eutherians, the Y chromosome is highly diverged between these two lineages and even between species, varying in size and gene order as well. Even between closely related species the Y chromosome can vary greatly in size. For instance, the chimpanzee Y is 24 Mb compared to the 60 Mb human Y chromosome [119]. The marsupial Y is much smaller, with an estimated size of ~10 Mb and representing a mere 1% of the haploid genome [120]. Comparisons of gene content between marsupial and eutherian Y chromosomes would therefore provide great insight into the evolution of this unusual chromosome.

Most genome projects only sequence a female of the species to obtain good sequence coverage of the X chromosome. This is true for the opossum [13] and tammar wallaby [10] but male devil genomes have been sequenced [11, 12]. However, no attempt has been made to assemble sequence for the devil Y chromosome. One of the confounding factors with sequencing Y chromosomes is its highly repetitive nature, making it essential to use a BAC-based approach [119, 121, 122]. In addition to using probes for three genes known to be on the tammar wallaby Y chromosome, a novel approach was developed for obtaining tammar wallaby Y-specific BAC clones in which Y chromosome probes were isolated by flow cytometry or by manual microdissection and hybridised to BAC library filters, creating a sublibrary enriched for Y-specific BACs [123, 124]. The sequencing of 10 Y-specific BAC clones led to the discovery of five previously unknown Y genes (*RPL10Y, MECP2Y, HCFC1Y,and HUWE1*) in addition to five known Y genes (*SRY, RBMY, KDM5D, UBE1Y, and ATRY*) [124]. All ten genes have a partner on the X chromosome. Orthologues of these genes were detected in the devil testis transcriptome, suggesting that the marsupial Y chromosome is conserved [124]. All except two of the marsupial Y-borne genes are ubiquitously expressed in the tammar wallaby [124]. The exceptions are *ATRY, *expressed exclusively in testis [124, 125], and *RMBY, *which is predominately expressed in testis but has very low levels of expression in kidney, lung, and spleen [124, 126].

More genes from the YCR (corresponding to the XCR) are present on the marsupial Y than on the Y of eutherians and those present on the marsupial Y have low nonsynonymous (Ka) to synonymous (Ks) substitution rates. Thus, marsupial Y genes are under purifying selection, perhaps as a consequence of them acquiring male-specific functions. In fact, some of these marsupial-specific Y genes are considered to be excellent candidates for functions in early development of male marsupials [124]. For instance, *ATRY* is expressed throughout testicular differentiation in the tammar wallaby, whereas its gametologue,* ATRX, *is expressed during development of the brain, neural tube, dorsal ganglia, and limbs [125]. Future research will undoubtedly address the function of more of these novel Y genes. 

### 6.3. X Chromosome Inactivation in Marsupials

Studies of X chromosome inactivation in marsupials have benefitted greatly from marsupial genome projects. Decades old isozyme studies on the inactivation status of just three genes (*G6PD, GLA, and PGK*) revealed that, like eutherians, marsupials inactivate one X chromosome in females (reviewed in [127, 128]). However, contrary to the random X inactivation observed in human and mouse, biochemical and replication timing studies revealed that the paternally derived X is preferentially silenced in marsupials [129–131]. Considerable variation in the extent of inactivation was observed between the three genes (*G6PD, GLA, and PGK1*) used for these studies, as well as between tissues and species, making it difficult to draw conclusions about the nature of marsupial X chromosome inactivation [127, 128]. The availability of assembled genomes and next-generation sequencing technology makes it now possible to confirm whether paternal X inactivation is observed for many more genes than the three mentioned above. This will hopefully be carried out in the near future. 

Direct determination of the inactivation status of other genes on marsupial X chromosomes was not possible until the sequence of marsupial X chromosomes was obtained. An activity map of the tammar wallaby X chromosome was constructed by examining the expression of 32 genes distributed across the X chromosome using RNA-FISH on fibroblast nuclei. This map shows that no gene is expressed from only one X chromosome in every cell but that all genes have a proportion of nuclei (5–68%) displaying biallelic expression (Figure 3) [132]. Comparable results were found for 12 genes in opossum and four genes in devil fibroblasts [133]. Unfortunately, RNA-FISH is unable to distinguish the maternal and paternal alleles but it is assumed that in nuclei showing monoallelic expression it is the maternal copy that has been detected. Therefore, the partial expression of the paternal X observed in the early biochemical studies is most likely the result of a mosaic cell population, where some cells express both alleles and others express just one [132]. 

Two alternative hypotheses had been proposed to explain the differences observed between genes and tissues in the extent of inactivation in marsupials. One hypothesis suggested that the silencing of loci on the X chromosome is a chromosome-wide phenomenon, with inactivation spreading from an inactivation centre to result in a correlation between the level of silencing and position on the X chromosome relative to the inactivation centre [134]. Genes closest to the inactivation centre would be subject to more complete silencing than those further away. The discovery of the candidate marsupial-specific inactivation centre *RSX *[118] could perhaps have given some credence to this idea. The alternative posits that marsupial X inactivation is regulated in small domains, rather than across the entire chromosome [135]. The activity map of the tammar wallaby X chromosome clearly shows no correlation between gene location and the extent of silencing, thereby refuting the former hypothesis [132]. Moreover, examination of two loci in close proximity to one another on the chromosome and in nuclei displaying monoallelic expression of both loci indicated that expression is coordinated from a single X chromosome [132]. However, Al Nadaf et al. [132] found that expression from the “inactive” X was discordant, conflicting with the idea of small domains of coordinate control. Instead, it appears that expression from the “inactive X” is determined on a gene-by-gene basis. 

The early isozyme studies found that X inactivation in fibroblasts was generally not as tightly controlled as that observed in somatic tissues. Indeed, RNA-FISH experiments on seven genes from different locations on the opossum X chromosome showed a much higher frequency of inactivation, with monoallelic expression detected in 96–100% of cells [136]. Unfortunately, none of these genes corresponded to those tested in fibroblasts, making it difficult to draw conclusions as to whether somatic tissues generally display tighter inactivation or if this is a feature of this selected subset of genes. 

The silenced status of the X chromosome is maintained in eutherians by a series of epigenetic modifications, including the accumulation of histone marks associated with repression of transcription, loss of marks associated with active chromatin, and methylation of 5′ CpG sites [137]. The depletion of marks associated with active chromatin is also a feature of marsupial XCI [138–140], as is a transient enrichment of the repressive mark H3K27me3 [136, 141]. In contrast, no evidence of differential methylation of CpG islands between active and inactive X chromosomes has been detected in marsupials in the two genes that have been examined to date [142–144]. The current availability of techniques able to detect differential methylation on a genome-wide scale will permit a more thorough investigation of the role of DNA methylation in marsupial XCI at 5′ CpG sites.

Since its initial discovery by Mary Lyon over 50 years ago [145], it has been assumed that X chromosome inactivation evolved as a mechanism to balance the expression of X-borne genes between the sexes. This idea was supported when the level of G6PD activity in erythrocytes of several members of the kangaroo family was examined, revealing equal levels of enzyme activity between females and males, but did not for G6PD activity levels in culture fibroblasts, where females had up to twice the activity of the male counterparts [146]. This question was not addressed again until sequence information for X-linked genes became available. Examination of female to male expression ratios for 12 genes showed a wide range of female to male ratios, varying from a ratio of 1 (indicative of complete dosage compensation) to 3 (no compensation) which was to some extent attributed to a considerable level of expression variation between individuals [132]. 

RNA-sequencing (RNA-seq) is enabling such dosage compensation studies to be examined across the entire X chromosome. Recently, RNA-seq data was obtained from five somatic tissues of a female and male opossum, revealing virtually complete dosage compensation for brain, cerebellum, kidney, and liver. Heart was the only tissue with a slight yet significant deviation from complete dosage compensation between the sexes [147]. Therefore, efficient dosage compensation is observed for opossum somatic tissues, which corresponds to the tightly controlled inactivation observed for brain and liver by RNA-FISH [136]. It is hoped that this type of global approach to examining dosage compensation will be carried out on more marsupials and with larger sample sizes. The inclusion of culture fibroblasts in these studies, which appears to have quite a different level of inactivation, would also be worthwhile. 

## 7. Evolution of Genomic Imprinting

Genomic imprinting is an epigenetic phenomenon where alleles are expressed in a parent-of-origin fashion. Marsupial X chromosome inactivation was actually the first reported example of genomic imprinting in mammals [130, 131]. Epigenetic modifications, such as CpG methylation and/or histone modifications, mark the silenced allele. The evolution of such a mechanism seems counterintuitive, as it leaves no backup copy in the case of a deleterious mutation in the expressed allele. Hence, many questions have been raised regarding its evolution. It does appear to be linked to the evolution of viviparity in mammals as no evidence of genomic imprinting has been detected in the egg-laying monotremes [148]. By examining the orthologues of loci imprinted in eutherians in marsupial genomes, it becomes possible to start addressing some of the questions surrounding its evolution. 

The first gene reported to be imprinted in marsupials was *IGF2*, a gene that is expressed from the maternal allele [149]. Imprinted expression of *IGF2* in the tammar wallaby appears to be controlled in a tissue- and developmental-specific fashion, similar to that observed in eutherians. For instance, although *IGF2* expression was detected throughout the placenta, it was only imprinted in the vascular and trilaminar region, and in liver it switched from being imprinted in pouch young to being biallelically expressed in adult liver [150]. As imprinted expression of this gene in eutherians is dependent on a differentially methylated region [151, 152], searches for such a region controlling the marsupial *IGF2* locus were performed. Initial searches failed to identify such a region [153], perhaps hampered by an abundance of low complexity polynucleotide repeats occurring at this locus [150]. More extensive searches for allele-specific methylation patterns eventually led to the identification of a differentially methylated region, demonstrating that, like eutherians, imprinting of the *IGF2* locus in marsupials is dependent on differential methylation. 

An imprinted gene located near *IGF2* in humans is the paternally expressed long non-coding RNA *H19. *The poor level of sequence conservation typical of non-coding RNAs hindered the identification of this gene in marsupials and it was initially thought that *H19* was absent [154]. Sequencing of three tammar wallaby BAC clones spanning the *IGF2/H19* locus and sensitive sequence similarity searches identified a putative *H19 *orthologue with 51% similarity to human [155]. Marsupial *H19* is expressed only from the maternal allele. Methylation of three sites upstream of *H19* was observed on the paternal copy, originating in the male germline. Sites of this differential methylation correspond with CTCF binding motifs, with the methylation of these sites functioning as transcriptional insulators [155].

The imprint status of over 20 genes has now been determined in marsupials (Table 2). Less than half of these show an imprinted mode of expression in marsupials, suggesting that genomic imprinting arose on a gene-by-gene basis. Finding one marsupial orthologue from a eutherian imprinted gene cluster does not mean that all genes within the cluster in marsupials are imprinted. For example, the *PEG10* locus located on human chromosome 7q21 contains five imprinted genes: *PEG10* and *SGCE* are expressed from the paternal allele, whereas *CALCR, TFP12, *and *PPP1R9A* are expressed only from the maternal allele in certain tissues [169]. As in human and mouse, *PEG10 *in marsupials  is expressed almost exclusively from the paternal allele. However, *SGCE *and* PPP1R9A* were biallelically expressed, but *SGCE *did show evidence of preferential expression from the paternal allele [166]. Imprinting of genes in this region is dependent on methylation of a putative imprint control region (ICR) within the promoters of *PEG10* and *SGCE.* There is conservation of the CpG island ICR region and differential methylation in marsupials [166]. Comparative genomic analysis ascertained that *PEG10* is derived from a sushi-ichi retrotransposon inserted into the region after the divergence of monotremes and therian (marsupial and eutherian) mammals and imprinting spread to more genes in the eutherian lineage [166].

Comparative genomics has proven valuable for understanding why many genes imprinted in eutherian species are not imprinted in marsupials. Genes within the Callipyge locus, *DIO3, DLK1,* and *RTL1*, are imprinted in eutherians but no evidence of an imprinted mode of expression has been detected in marsupials [160, 162]. The genomic landscape of this locus has changed considerably during mammalian evolution, expanding to twice the size of the eutherian locus due to an accumulation of LINE1 elements. In eutherians, SINE repeats have been selected against and there is an increase in GC and CpG island content. Phylogenetic footprinting revealed over 140 evolutionary conserved regions yet none of these match the known eutherian imprint control region, consistent with the lack of imprinting at this locus in marsupials. A retrotransposition event appears to have led to the formation of a new gene in eutherians, which may have been responsible for driving the evolution of imprinting at this locus [160]. 

The Prader-Willi/Angelman syndrome locus is an imprinted domain on human 15q11-q13 where mutations often lead to the neurological disorders Prader-Willi and Angelman syndrome. Imprinting in this region is controlled by an imprint control region, situated between paternally expressed *SNRPN* and the maternally expressed *UBE3A *gene [170]. A comparison of the gene arrangement for this locus across vertebrates led to an unexpected finding. In marsupials, *SNRPN* and *UBE3A* are located on different chromosomes, with *UBE3A* located adjacent to *CNGA3*, a human chromosome 2 gene [165]. This arrangement of *UBE3A *and *CNGA3* was the same in platypus, chicken, and zebrafish genomes, indicating that it is ancestral. Interestingly, *SNPRN* only exists in therian species and probably arose by tandem duplication of *SNRPB* [165, 171], a nonimprinted gene located on human chromosome 20. Three intronless paternally expressed genes from this region (*NDN, MAGEL2,* and *MKRN3*) in eutherians are completely absent in all other vertebrates and most likely arose from retrotransposition events after the divergence of marsupials and eutherians [165]. Neither *SNPRN *nor *UBE3A* are imprinted in marsupials, suggesting that imprinting of these genes only occurred once this region had been assembled in the eutherian lineage [165].

Although there are a number of hypotheses proposed to explain the evolution of genomic imprinting, it is generally agreed that it is linked to the evolution of the placenta and viviparity [172]. One of the more popular hypotheses, the parental-conflict hypothesis, claims that imprinting arose from a conflict between maternal and paternal genomes over the provision of maternal resources [173]. An organ in which this would be especially evident is the placenta, as it is entirely fetally derived and is essential for the supply of nutrients and oxygen to the fetus. Indeed, the placenta has a high number of imprinted genes, mainly those involved in placental and fetal growth [172]. However, the marsupial mode of reproduction only relies on the placenta for a short period of time and has a much larger maternal investment in lactation. Is it then possible that genomic imprinting may be more prevalent in the mammary gland of marsupials [10]? 

Studies on the imprint status of two key genes required for the onset of lactation (*IGF2* and *INS*) in the tammar wallaby have revealed that they are indeed imprinted throughout lactation [174]. Finding these genes imprinted in mammary gland actually fits in better with an alternative hypothesis to explain the evolution of genomic imprinting, referred to as the maternal-infant coadaptation hypothesis, which claims that genomic imprinting arose from the intimate interaction between mother and offspring for genes involved in the regulation of the requirements and behaviour in the offspring, and the same genes involved in the regulation of the mother's response to her offspring [175]. Thus, future studies of genomic imprinting in marsupials should focus on the mammary gland [10, 174]. Next-generation sequencing technologies lend themselves to the identification of novel imprinted genes and could lead to the rapid identification of marsupial-specific-imprinted genes, if any exists [175]. 

## 8. Marsupial Genome Evolution

One remarkable feature of marsupial genomes is the high degree of chromosome conservation, which contrasts the extensive chromosomal rearrangement observed amongst eutherians. From the earliest karyotyping studies, determining chromosome number and morphology, to later studies using G-banding and chromosome painting for species spread across marsupial phylogeny, it was evident that marsupial chromosomes had changed little since divergence from a common ancestor [50, 176–178]. Despite this astounding level of chromosome conservation, the diploid number of the marsupial ancestor has been much debated [15], but it has only been since the marsupials entered the genomics era that comparisons to outgroups have been possible to assist in resolving the ancestral chromosome arrangement. 

Diploid chromosome numbers in marsupials are bimodally distributed, with 2*n* = 14 and 2*n* = 22 complements prevalent both in Australidelphia and Ameridelphia [178, 179]. This led to the proposal of two alternative hypotheses for the chromosome number of the ancestral marsupial [176, 180–182]. The first posited a 2*n* = 14 ancestral karyotype, since very little difference in G-banding patterns was observed between representatives of different marsupial lineages [176]. A well-supported phylogenetic tree with chromosome numbers plotted on to it provided further support for a 2*n* = 14 ancestor [183]. The alternative hypothesis proposed that the higher diploid number of 2*n* = 22 was ancestral, with lower diploid numbers being the result of fusion events [180, 184]. This hypothesis was originally proposed based on its prevalence amongst marsupials [180], but chromosome painting showed that 2*n* = 22 karyotypes present in different species were not equivalent [50, 185]. Nevertheless, this did not eliminate a 2*n* = 22 ancestral karyotype that was subject to several fusion events very early in marsupial evolution to result in the common 2*n* = 14 karyotype. However, the only data presented to support this hypothesis is the presence of interstitial telomere signals (ITS) in Ameridelphia species with lower diploid numbers, where ITSs were taken as evidence of past fusion events [184]. Deciphering which hypothesis is more likely to be true could not be more conclusively resolved without reference to an outgroup.

**Figure 4:**
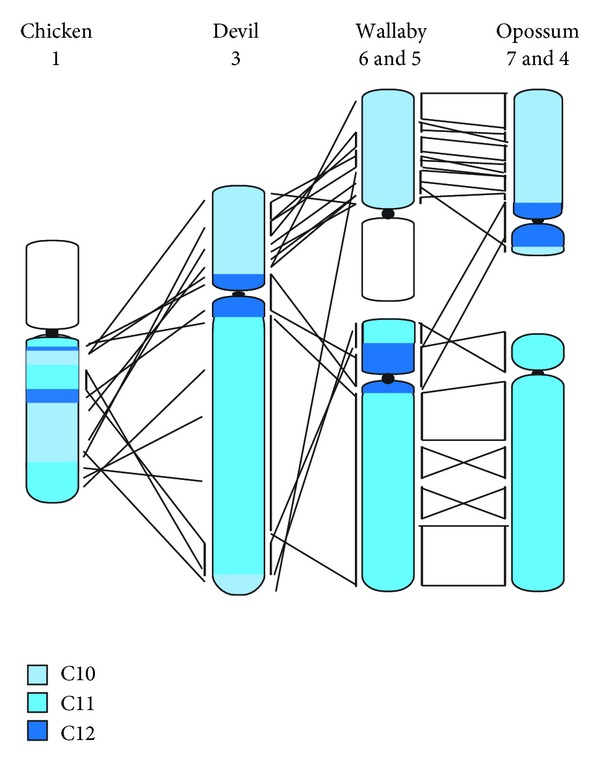
Arrangement of genes from conserved segments C10, C11, and C12 in chicken (outgroup) and marsupials. Most genes from these three segments are intermingled on chicken chromosome 1. They remain together on devil chromosome 3 but are distributed across two chromosomes both in the tammar wallaby and opossum, with independently derived breakpoints. Vertical lines indicated conserved blocks of genes.

Cross-species chromosome painting amongst marsupials showed that marsupial karyotypes can be divided into 19 conserved segments (referred to as C1–C19) [50]. The anchored opossum genome assembly has made it possible to predict the gene content of these segments and enabled comparisons of their arrangement to be made to outgroups such as chicken and human. The fragmented nature of the genome assemblies for the other two sequenced marsupials is less than ideal for this type of research but physical mapping of genes in these species has partially compensated for the reduced level of assembly quality [10, 27, 28, 33]. Comparisons of arrangement between all three species revealed substantial rearrangement, with some regions of the genome appearing to be particularly prone to genome restructuring events such as inversions, for example, C2, C3, and C4 [10, 33]. The segments displaying the most conserved gene order corresponded to segments C11 and C12 [33], which together with C10 make up devil chromosome 3, tammar wallaby chromosome 5 and 6, and opossum chromosomes 4 and 7 (Figure 4) [50]. If the marsupial ancestor had a 2*n* = 14 chromosome complement, segments C10, C11, and C12 would form a single chromosome as observed in the devil. Alternatively, a 2*n* = 22 ancestor sees these segments distributed between two chromosomes [183]. In the chicken, genes from all three segments are for the most part found on chicken chromosome 1, suggesting that they were syntenic in the ancestor of all mammals and remaining as a single chromosome in extant 2*n* = 14 marsupials [28]. This refutes the 2*n* = 22 ancestor hypothesis. 

What about those ITSs? These signals, detected in a number of marsupials, colocalise with constitutive heterochromatin and therefore, are likely to be a component of satellite DNA [186–188]. Furthermore, comparing the location of ITS with marsupial chromosome homology maps clearly shows that many ITSs would not correspond to past fusion events. For instance, ITSs detected in the pericentric regions of chromosomes for two other members of the Dasyuridae family, both with 2*n* = 14 karyotypes, are present on chromosomes 1, 2, 3, and 6 [188]. Assuming these species were derived from a 2*n* = 22 ancestor, chromosome 1 would be the result of the fusion of two chromosomes; one consisting of conserved segments C1–C3 and the other, segments C4–C6. However, these *Sminthopsis* species have experienced two inversions [189]; meaning that the detection of telomeric remnants at the centromere would be unexpected [15]. Likewise, ITS detected at the centromere of chromosome 6 [188] would not correspond to the site of a past fusion event, as this chromosome would have been a single chromosome in the predicted 2*n* = 22 ancestral karyotype [15].

Thus, all evidence, including extensive G-banding studies, cross-species chromosome painting, phylogenetics, and genome comparisons, support the 2*n* = 14 ancestor hypothesis, similar to the 2*n* = 14 karyotypes observed in extant species from six of the seven marsupial orders. 

## 9. Genome Sequencing and Devil Facial Tumour Disease

The Tasmanian devil has suffered a major population crash and is threatened with extinction in the wild in the next few decades as a result of a transmissible cancer [32]. DFTD is most unusual as it appears to be the tumour cells themselves that are the infectious agent being spread by biting [190]. Biting behaviour frequently occurs during communal feeding and mating. The tumour cells transmitted between individuals are able to evade detection by their immune system and grow unimpeded. DFTD appears to have a 100% mortality rate. Devils infected with DFTD often die from starvation as a consequence of the facial tumours making feeding difficult, from organ failure or secondary infection [191]. Genomic approaches have been used to rapidly gain an understanding of this terrible disease, working towards the identification of diagnostic markers, effective treatments, or the development of a vaccine [9]. 

The allograft theory of DFTD transmission was first proposed after karyotype analysis demonstrated that tumours from 11 different individuals were identical yet had been subject to complex rearrangement [190]. This karyotype consisted of 13 chromosomes (the normal devil karyotype is 2*n* = 14), with both chromosome 1s, one homologue of chromosome 6, and the sex chromosomes unrecognisable in G-banded karyotypes and there was four marker chromosomes of unknown origin [190]. This theory was subsequently supported by MHC allele [52] and microsatellite typing [52, 192], mitochondrial DNA sequencing [11, 12], and single nucleotide polymorphism (SNP) typing [12], all of which showed that the genotypes of tumours were identical or very similar and different to that of their hosts. Since the first cytogenetic study was carried out, four new karyotypic “strains” of the tumour have been identified, all of which appear to be derived from the same tumour and indicate that the tumour is evolving as it spreads through the population [193]. Differences in growth rates have been observed between these strains, with strain 2 having the slowest growth in culture and longer survival period in infected devils [194]. Strain 4, the chromosomally most derived karyotypic strain, has the fastest growth rate and may be more virulent due to the presence of a variable number of double minute chromosomes [193], which in humans are often associated with oncogene amplification and more aggressive malignancies [195]. 

Traditional microscopic techniques struggled to identify the tissue origin of the DFTD tumour and simply referred to it as a poorly differentiated soft-tissue neoplasm [196]. Immunological staining suggested that it was of neuroendocrine origin [197]. Sequencing the transcriptome of the tumour finally solved this mystery. The expression profile of the tumour matched that of a myelinating cell. More specifically, the detection of the gene encoding for periaxin protein (*PRX*), a Schwann cell-specific gene, indicated a Schwann cell origin [192]. All DFT cells have since been shown to have intense staining for periaxin by immunohistochemistry, thereby providing a diagnostic maker for DFTD [192, 198]. 

Since DFTD appears to be derived from a tumour that arose in one individual and then spread through the population, an obvious first step to understanding DFTD is to characterise the genomic changes that have occurred in the tumour. Most known genes associated with cancer are tumourigenic as a consequence of genome rearrangements that result in the formation of fusion genes, copy number variations, or alter the transcriptional regulation of genes [199]. Mutations driving tumourigenesis must therefore affect key pathways. Uncovering such perturbed pathways in DFTD requires information on genomic rearrangement [9]. The latest advances in cancer genome sequencing in humans permit a genome-wide survey of all rearrangements at unparalleled resolution [200]. These next-generation sequencing approaches are highly sensitive and provide resolution at the base pair level on breakpoint rearrangements and mutations [201]. All types of mutations, including deletions, SNPs, and small insertion-deletions (indels), can be identified with these sequencing approaches [202]. However, the most important resource for these studies is a good reference genome, one that has been deeply sequenced, and just as importantly, anchored to chromosomes.

In addition to the devil genome sequence projects outlined previously, three DFTD tumours have been sequenced in order to detect potential driver mutations for tumourigenesis [11, 12]. Two primary tumours were sequenced from individuals captured in the southeastern region of Tasmania (Forestier Peninsula), while the other was a lung metastasis from a devil captured on the north coast [11, 12]. Tumour sequences have flagged several genes with mutations causing amino acid substitutions as candidate driver mutations. Among these are *ANTXR1*, a regulator of the infamous *TP53* gene often referred to as the “guardian of the genome” [12], *RET* which is a protooncogene [11], and *FANCD2 *[11], a member of the Fanconi anemia family important for genome stability [203]. Three candidate cancer-associated metabolic pathways have been put forward due to nonsynonymous mutations in the genes *PRHCK, GALNS,* and *CCNA-*like [12]. At least two genes have been completely deleted from the DFTD genome (*MAST3* and *BTNL9*) but no in-frame fusion genes have been detected [11]. 

The limitation of these sequencing efforts is a lack of a well-assembled and anchored reference genome, making it more difficult to accurately detect structural mutations. The best assembly consisted of well over 30,000 unordered scaffolds on devil chromosomes. Breakpoints in a primary DFTD tumour and in a lung metastasis have been identified and verified, which provides some idea of the structural rearrangements that have occurred [11]. Nonetheless, without a reference assembly ordered on devil chromosomes, it remains difficult to accurately determine the extent of genomic restructuring that has taken place and the new genomic context in which this places candidate genes potentially involved in tumourigenesis. Molecular cytogenetics techniques have been used to complement the sequencing data. Chromosome painting was used to detect gross homology between normal and DFTD chromosomes, and gene mapping to detect rearrangements on a finer scale [33]. These techniques have also been used to gain insight into the differences between karyotypic strains and tumour evolution. 

**Figure 5:**
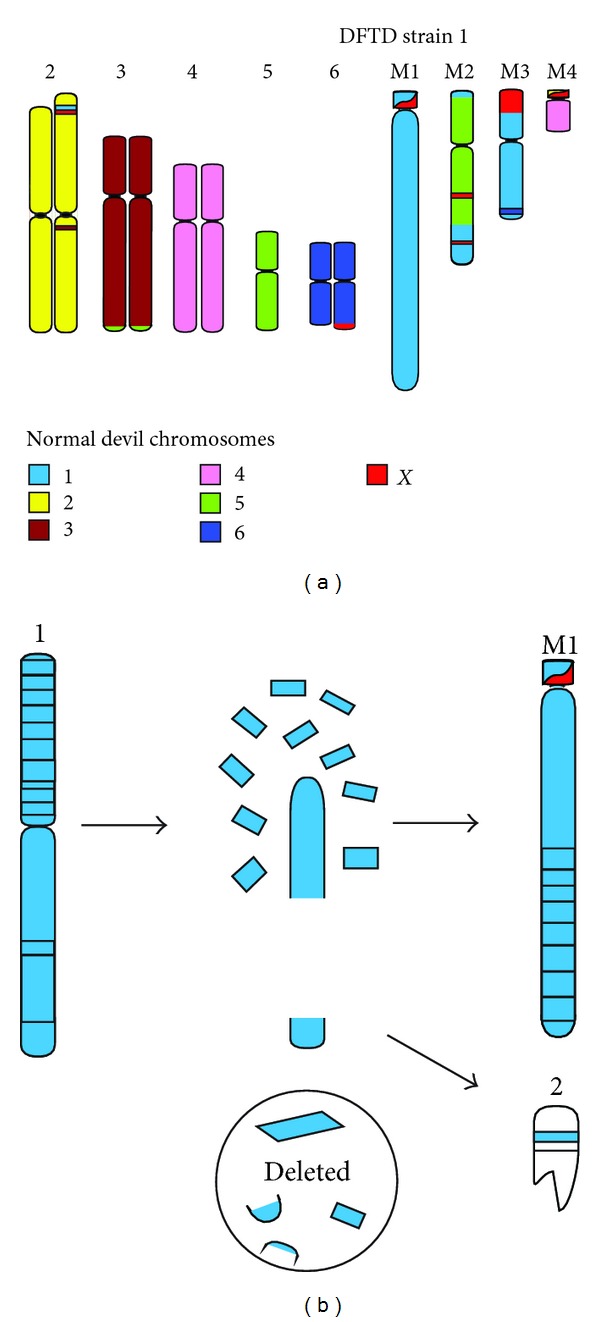
(a) Schematic of DFTD strain 1 karyotype. Chromosomes have been colour coded to reflect their homology to normal devil chromosomes (modified from [33]). (b) One homologue of chromosome 1 appears to have been shattered by a chromothripsis event. Some chromosome fragments have been lost, but most rejoin to form marker chromosome 1 (M1).

Chromosome painting showed that the marker chromosomes of strain 1 through 3 largely consisted of chromosome 1, 5, and X material, which was supported by mapping of over 100 genes by fluorescence *in situ* hybridisation onto the same strains (Figure 5(a)). The painting and mapping data demonstrated that, despite passage through over 100,000 individuals, DFTD chromosomes have remained remarkably stable [33]. Chromosome 1 material appears to be particularly stable with no differences detected in the order of 52 genes from this chromosome between the three tumour strains, suggesting that rearrangement of chromosome 1 may have been the initial step in the development of DFTD and that the maintenance of this arrangement is required to preserve the tumourigenic properties of a DFTD cell [33, 204]. Differences identified between DFTD strains were restricted to well-demarcated regions of the genome, predominantly consisting of chromosome 4, 5, and X material [33].

The original DFTD tumour may not have arisen from the traditionally accepted gradual accumulation of mutations model, as the extensive chromosomal rearrangements observed resemble those recently proposed to occur by a single cataclysmic event referred to as chromothripsis. This is a phenomenon where either a chromosome segment or even several chromosomes are shattered and reformed into a chromosome(s) by the nonhomologous end-joining DNA repair mechanism, manifesting as extensive rearrangement of only certain regions of the genome [205]. Signatures of chromothripsis are observed in the DFTD tumours, with extensive rearrangement limited to just a few chromosomes [33], very little variation in copy number [11, 33], and evidence of microhomology-mediated end-joining [11]. One homologue of chromosome 1 shows a particularly rearranged gene order, appearing to have been shattered into at least 16 pieces and rejoined to form the distinctive marker 1 (M1) chromosome (Figure 5(b)). 

Genomic resources for the devil have permitted knowledge of DFTs to be rapidly acquired, but there is still much left to be done. Further integration of the tumour sequence data with cytogenetic mapping is required to home in on candidate genes and pathways driving tumourigenesis as well as tumour evolution. The extensive structural rearrangement [33], the proposed chromothripsis mechanism for the generation of the tumour genome [9, 33], and the paucity of nonsynonymous mutations identified by sequencing [11, 12] would strongly suggest that future studies need to focus on accurately identifying structural mutations. 

## 10. Conclusions

Marsupial genome sequences have greatly accelerated research on some of the unique features of this mammalian lineage. Here I have highlighted just several areas that have particularly forged ahead since the release of the first marsupial genome assembly. 

The identification of highly divergent immune genes laid to rest the idea of the marsupial immune system being inferior to that of eutherians. In fact, it appears that marsupials could be a potential source of potent antimicrobials, particularly those present in milk [62, 63, 93]. The reproductive strategy of marsupials lends itself to studies of lactation and development. Continued research in marsupials on *HOX* genes and other genes involved in the development will be useful for elucidating how morphological diversity is achieved. Marsupial sex chromosomes have been the source of some very unexpected findings, like the absence of the *XIST* gene [115–117], which had been the subject of extensive research for over 20 years, and the recent discovery of a marsupial-specific *XIST-*like gene [118]. The sequencing of novel marsupial Y chromosome genes has greatly impacted on our understanding of Y chromosome evolution in therian mammals [124]. Progress towards unravelling the evolutionary origin of genomic imprinting has proceeded at a rapid rate over the last several years, revealing fewer imprinted genes in marsupials than in eutherians. However, perhaps research in this area has been led astray by looking only at those genes imprinted in eutherians. With a greater maternal investment in lactation in marsupials, it makes sense to look for imprinted gene expression in the mammary gland [10, 174]. A comparison of gene arrangement in marsupials compared to outgroups has finally resolved the long-standing debate over the diploid chromosome number of the ancestral marsupial and allowed its karyotype to be reconstructed [15]. 

Perhaps the best example of the utility of marsupial genome sequence is the rapid progress that has been made towards understanding the devastating disease threatening Australia's iconic Tasmanian devil. Since the first publication positing the allograft theory of transmission [190], many questions have been answered through genomic approaches. With a proposed increase risk of diseases emerging in the future, it is hoped that the knowledge we have gained from marsupial genome projects will help to ensure the survival of these amazing animals. 

## Figures and Tables

**Table 1:** There are currently 63 Australian marsupials listed as threatened on the IUCN list.

Order	Extinct	Extant	Threatened	Near threatened
Dasyuromorphia (e.g., devil, quolls, dunnarts)	1	73	12	10
Diprotodontia (e.g., wombats, wallabies, possums)	7	139	45	16
Notoryctemorphia (marsupial mole)*	0	2		
Peramelemorphia (e.g., bandicoots, bilby)	3	19	6	1

*Data deficient—threatened status not determined.

**Table 2:** Orthologues of eutherian imprinted genes and their imprint status in marsupials.

Imprinted genes			Nonimprinted genes		
Gene	Expressed allele	References	Gene	Present/Absent in genome	References
*H19 *	Paternal	[155]	*CDKN1C *	Present	[156, 157]
*HTR2A *	Maternal	[158]	*COPG2 *	Present	[158]
*IGF2 *	Paternal	[149, 150, 156, 159]	*DIO3 *	Present	[160]
*IGF2R *	Maternal	[161]	*DLK1 *	Present	[160, 162]
*INS *	Paternal	[163]	*GRB10 *	Present	[164]
*L3MBTL *	Paternal	[158]	*IMPACT *	Present	[158]
*MEST *	Paternal	[156, 158]	*MAGEL2 *	Absent	[165]
*PEG10 *	Paternal	[166]	*MEG3 *	Absent	[162]
			*MKRN3 *	Absent	[165]
			*NDN *	Absent	[165]
			*NNAT *	Absent	[167]
			*PHLDA2 *	Present	[168]
			*PLAGL1 *	Present	[158]
			*SGCE *	Present	[166]
			*SNRPN *	Present	[165]
			*UBE3A *	Present	[165]
